# Predicting red blood cell transfusion in hospitalized patients: role of hemoglobin level, comorbidities, and illness severity

**DOI:** 10.1186/1472-6963-14-213

**Published:** 2014-05-10

**Authors:** Nareg H Roubinian, Edward L Murphy, Bix E Swain, Marla N Gardner, Vincent Liu, Gabriel J Escobar

**Affiliations:** 1 Blood Systems Research Institute, 270 Masonic Avenue, San Francisco, CA 94118, USA; 2 Division of Research, Kaiser Permanente Northern California, 2000 Broadway Avenue, Oakland, CA 94612, USA; 3 Department of Laboratory Medicine, University of California, 270 Masonic Avenue, San Francisco, CA 94118, USA

## Abstract

**Background:**

Randomized controlled trial evidence supports a restrictive strategy of red blood cell (RBC) transfusion, but significant variation in clinical transfusion practice persists. Patient characteristics other than hemoglobin levels may influence the decision to transfuse RBCs and explain some of this variation. Our objective was to evaluate the role of patient comorbidities and severity of illness in predicting inpatient red blood cell transfusion events.

**Methods:**

We developed a predictive model of inpatient RBC transfusion using comprehensive electronic medical record (EMR) data from 21 hospitals over a four year period (2008-2011). Using a retrospective cohort study design, we modeled predictors of transfusion events within 24 hours of hospital admission and throughout the entire hospitalization. Model predictors included administrative data (age, sex, comorbid conditions, admission type, and admission diagnosis), admission hemoglobin, severity of illness, prior inpatient RBC transfusion, admission ward, and hospital.

**Results:**

The study cohort included 275,874 patients who experienced 444,969 hospitalizations. The 24 hour and overall inpatient RBC transfusion rates were 7.2% and 13.9%, respectively. A predictive model for transfusion within 24 hours of hospital admission had a C-statistic of 0.928 and pseudo-R2 of 0.542; corresponding values for the model examining transfusion through the entire hospitalization were 0.872 and 0.437. Inclusion of the admission hemoglobin resulted in the greatest improvement in model performance relative to patient comorbidities and severity of illness.

**Conclusions:**

Data from electronic medical records at the time of admission predicts with very high likelihood the incidence of red blood transfusion events in the first 24 hours and throughout hospitalization. Patient comorbidities and severity of illness on admission play a small role in predicting the likelihood of RBC transfusion relative to the admission hemoglobin.

## Background

Variation in clinical transfusion practice has long been recognized, particularly among acutely ill hospitalized patients [1-4]. Several well-powered randomized controlled clinical trials of certain groups of adult medical and surgical patients support the notion that restrictive red blood cell (RBC) transfusion strategies result in similar or better patient outcomes compared to a more liberal strategy [5-10]. These studies have led to the development of guidelines that endorse transfusion at lower hemoglobin levels during the perioperative period and intensive care unit stay [11-14].

However, integration of guidelines into practice has been variable, with a significant proportion of RBC units transfused outside of evidence-based indications [15-18]. It has been hypothesized that clinicians modify their transfusion decisions by incorporating patient comorbidities or severity of illness [15,16]. These factors might partially explain observed variation in RBC transfusion practice.

We describe the relationship between patient factors and RBC transfusions in the acute care community hospital setting, taking advantage of the existing research infrastructure of an integrated health care delivery system, Kaiser Permanente Northern California (KPNC). Using data from a comprehensive electronic medical record and an externally validated risk adjustment methodology applicable to all hospitalized patients, we quantified the incremental effect of increasing clinical detail on the likelihood of a patient receiving a RBC transfusion during hospitalization [19-21]. We sought to assess the role of patient comorbidities and severity of illness, in addition to hemoglobin levels, in predicting inpatient RBC transfusion events.

## Methods

This study was approved by the KPNC Institutional Review Board for the Protection of Human Subjects, which has jurisdiction over all the hospitals included in this study, and the University of California, San Francisco Committee on Human Research.

We performed a retrospective cohort study of all hospitalized non-obstetric patients age ≥18 years admitted to 21 KPNC hospitals between January 1, 2008 and December 31, 2011. The study hospitals have been described in previous reports and this cohort was recently employed for the development of a comprehensive risk adjustment system [19,20].

KPNC serves a total population of approximately 3.3 million members. All KPNC hospitals and clinics employ information systems linked by a common medical record number. We obtained data regarding use of blood products from the KPNC blood bank and audited a random sample of inpatient transfusion events to validate the accuracy of this record. The audit did not identify any transfusion events not present in the blood bank record (i.e., our search strategy had 100% specificity) and verified that 97% of blood products released from the blood bank were transfused (Additional file 1).

### Analytic approach

The methods we employed to retrieve, clean, and process other study data have been described elsewhere [19,20]. The principal (dependent) outcome for our analyses was whether or not a patient received a RBC transfusion either within 24 hours of admission to the hospital or ever during the hospitalization. We grouped predictor variables into six broad categories, so that each category incrementally added more clinical data. They included: ***administrative data*** (age, sex, comorbidity burden, emergency or elective presentation, medical or surgical admission, and admission diagnosis); ***admission hemoglobin*** (adding pre-hospital entry hemoglobin); ***severity of illness*** (adding illness severity); ***prior transfusion*** (adding history of a prior inpatient RBC transfusion within the past year); ***initial hospital location*** (adding the first unit of hospital entry) and ***hospital*** (adding individual hospitals as fixed effects).

Emergency versus elective presentation was based on whether patients were admitted after being evaluated in the emergency department. Admission diagnoses were based on International Classification of Diseases, 9^th^ Revision (ICD-9) diagnosis codes. Health Care Utilization Project (http://www.ahrq.gov/data/hcup) single-level diagnosis categories were used to organize all possible ICD admission codes into groups, with subsequent designation of five medical and surgical categories into Primary Conditions: Gastrointestinal Bleeding, non-surgical Cardiovascular, Infection, Malignancy, and Orthopedic Surgery (Additional file 1). These groupings of common conditions were chosen on the basis of their association with medical or surgical bleeding (Gastrointestinal Bleeding & Orthopedic Surgery), anemia of chronic illness (Malignancy & Infection), as well as the potential benefit of improving oxygen delivery with RBC transfusion (non-surgical Cardiovascular).

Admission hemoglobin was defined as the lowest hemoglobin within 72 hours prior to hospital entry from the emergency room or outpatient clinic or the most recent hemoglobin within 30 days prior to hospitalization for elective admissions. Initial hospital locations included the medical-surgical wards, intensive care unit, intermediate care areas, or operating room. Comorbid disease burden was quantified by a previously described continuous score, COPS2 (Comorbidity Points Score, version 2), which is based upon patients’ medical diagnoses for the 12 months preceding hospitalization [19]. For comparison purposes, we also employed the methodology of Deyo et al. to assign Charlson scores [22]. Severity of illness was quantified using a continuous score, LAPS2 (Laboratory Acute Physiology Score, version 2), a physiology-based score of individual laboratory test results, vital signs, and neurologic status, obtained within 72 hours prior to hospital entry [19]. We emphasize that admission hemoglobin was used for predictive modeling purposes, because it was commonly available on most patients at a comparable time point.

### Statistical methods

Categorical variables were summarized as frequencies and percentages and continuous variables as mean ± standard deviation. Continuous variables were grouped into quartiles (COPS2, LAPS2, age at admission) or into ranges for hemoglobin (<7, 7-7.9, 8-8.9, 9-9.9, ≥10 g/dL). We used the Kaplan-Meier method for depicting the timing of first RBC transfusion to account for censoring at the time of hospital discharge or death. We used multivariable logistic regression to build our predictive models including each variable category in an incremental fashion. We evaluated model performance with Nagelkerke’s Pseudo-R [2] and area under the receiver operator characteristic curve (C-statistic) for all patients and those with the five Primary Conditions. To further assess the effect of additional model components, we employed the methods described by Pencina et al to calculate the integrated discrimination improvement (IDI) and net reclassification improvement (NRI) [23]. These two indices have been shown to be useful in quantifying the effect size of an added predictor to a model, as they rely on the strength of a predictor’s association with the outcome and less on the strength of the baseline model [24]. Statistical analyses were performed in Stata 11 (Stata SE, Version 11.2, StataCorp, College Station, TX).

## Results

The study dataset included 444,969 hospitalizations involving 275,874 patients. RBC transfusions occurred in 32,493 patients (11.8%) and 61,988 hospitalizations (13.9%). Of these events, approximately half (6.0% of patients and 7.2% of hospitalizations) occurred within 24 hours of admission. Table 1 shows that, compared with the non-transfused cohort, the transfused cohort had higher illness severity, comorbidity burden, hospital length of stay, inpatient mortality, and 30-day mortality.

**Table 1:** Patient characteristics

**Patient Characteristics**	**Transfused**	**Not transfused**
No. patients/ no. hospitalizations	32,493 / 61,988	243,381 / 382,981
% male	43.7	45.8
Age^1^	69.1 (15.3)	63.7 (17.8)
% ≥ 65 years	65.4	51.3
LAPS2^1,2^	69.3 (44.1)	54.2 (38.2)
COPS2^1,3^	49.7 (45.1)	34.7 (37.5)
Charlson score (median, IQR)	2, 1 - 3	1, 0 – 2
Admission Hemoglobin^1,4^	9.9 (2.4)	12.9 (1.9)
% with these Primary Conditions^5^		
Gastrointestinal bleeding	11.5	1.4
Orthopedic surgery	10.9	4.6
Malignancy	9.5	5.7
Infection	11.8	13.1
Cardiovascular	6.2	11.5
Other Medical	33.6	43.7
Other Surgical	16.5	20.0
% not “full code” at time of admission	15.8	13.5
Hospital Length of Stay^1^	8.0 (12.2)	4.6 (4.3)
Mortality rate (%)		
In-hospital	6.1	2.5
30-day	8.7	4.6

Footnotes

^1^Mean (Standard Deviation).

^2^Laboratory Acute Physiology Score, version 2 (LAPS2); physiology-based score which includes vital signs, neurological status, and laboratory results.^19^ Increasing degrees of physiologic derangement and mortality are reflected in a higher LAPS2, which is a continuous variable that with a range between zero and 282 in this cohort.

^3^Comorbidity Point Score, version 2 (COPS2); a longitudinal, diagnosis-based score assigned monthly that employs all diagnoses incurred by a patient in the preceding 12 months.^19^ Increasing values of COPS2 are associated with increasing mortality with a range between zero and 306 in this cohort.

^4^Admission hemoglobin was available in 410,126 of 444,982 hospitalizations (92.0%).

^5^Primary Conditions are groupings of related International Classification of Disease codes assigned at the time of admission to the hospital. These codes are further grouped based on the schema used by the Agency for Healthcare Research and Quality’s Healthcare Cost & Utilization Project. See Additional file 1 for additional details.

Table 2 shows mean hemoglobin and RBC transfusion data for patients with the five Primary Conditions. Admission hemoglobin was not available for 34,843 hospitalizations (7.8%); the majority of these admissions (82%) were elective in nature, including elective surgery and chemotherapy. Admission hemoglobin was lowest for those with gastrointestinal bleeding (10.2 (2.7) g/dL) and highest in those admitted for orthopedic (13.3 (1.6) g/dL) and cardiovascular conditions (12.6 (2.1) g/dL). In the transfused cohort, the pre-transfusion hemoglobin was highest in individuals with cardiovascular (8.4 (1.4) g/dL) and orthopedic (8.4 (1.1) g/dL) admissions and the median time to the first RBC transfusion was shortest in those admitted with gastrointestinal bleeding (2 hours) and conditions related to malignancy (6 hours). Admission hemoglobin had a relatively linear relationship with pre-transfusion hemoglobin, especially in those transfused within the first 24 hours of hospitalization (Additional file 1: Table S1). The admission hemoglobin was within 1 g/dL of the first pre-transfusion hemoglobin in 60% of those transfused within 24 hours of hospitalization and in 40% of all transfused patients.

**Table 2:** Hemoglobin & transfusion characteristics

	**GI Bleed N=12,388**	**Infection N=57,473**	**Malignancy N=27,831**	**Cardiovascular N=47,996**	**Ortho surgery N=24,264**	**All admissions N=444,969**
Admission Hgb (ALL Patients)^1^	10.2 (2.7)	12.0 (2.1)	11.9 (2.8)	12.6 (2.1)	13.3 (1.5)	12.4 (2.2)
Admission Hgb (Transfused)^1^	8.6 (2.1)	9.4 (1.9)	8.7 (2.7)	9.6 (2.0)	12.3 (1.4)	9.9 (2.4)
Admission Hgb (Not Transfused)^1^	12.3 (1.9)	12.3 (1.8)	12.9 (1.9)	12.9 (1.8)	13.7 (1.3)	12.9 (1.8)
Hgb prior to RBC transfusion^1,2^	7.9 (1.5)	7.9 (1.1)	7.7 (1.8)	8.4 (1.4)	8.4 (1.1)	8.1 (1.5)
Patients transfused RBC (%)	7,099 (57)	7,320 (13)	5,884 (21)	3,869 (8)	6,785 (28)	61,988 (14)
Mean # of RBC ± SD	3.6 ± 3.0	2.7 ± 2.5	3.3 ± 2.7	2.6 ± 2.4	2.0 ± 1.2	2.9 ± 2.7
Time to transfusion, hours median^3^	2	26	6	22	43	23

Footnotes

^1^Hemoglobin (Hgb) value in g/dL (Standard Deviation). Admission hemoglobin was available in 410,126 hospitalizations (92.0%).

^2^Median time from pre-transfusion hemoglobin to RBC transfusion was 7 hours, IQR 3.5, 11.4 hours.

^3^Median time in hours from hospital admission to the first RBC transfusion.

**Figure 1:**
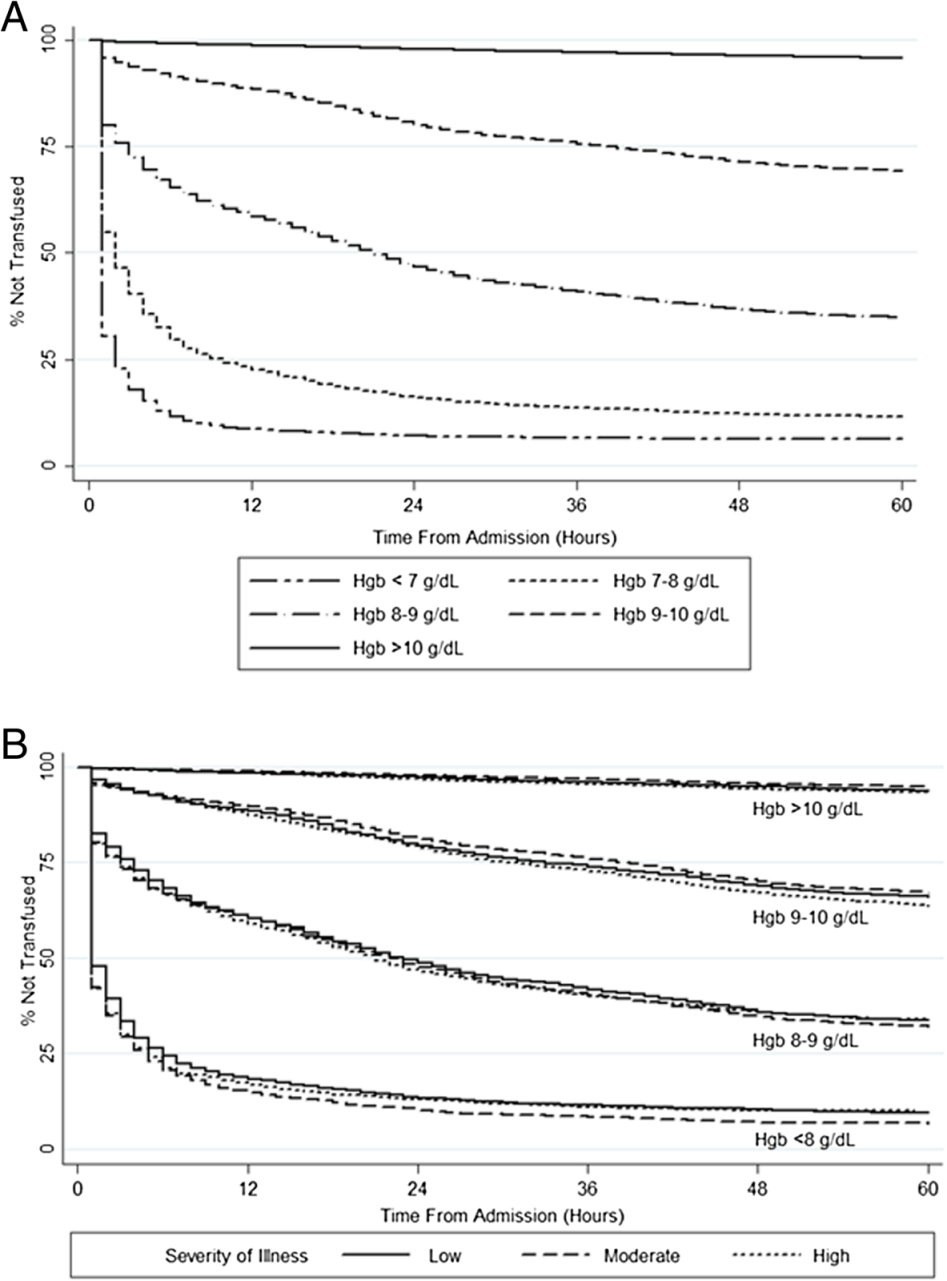
**Probability of Red Blood Cell Transfusion as a Function of Admission Hemoglobin and Severity of Illness. A)** The left panel shows that the likelihood of transfusion is tightly linked to the degree of anemia and that it falls exponentially in the first 24 hours, after which the rate of decrease is linear. **B)** The right panel shows that trends in severity of illness, within varying strata of admission hemoglobin, do not explain differences in overall rates of RBC transfusion. Severity of Illness refers to ranges of Laboratory Acute Physiology Score, version 2 (LAPS2) a physiology-based score which includes vital signs, neurological status, and laboratory results [19]. Increasing degrees of physiologic derangement are reflected in a higher LAPS2. Ranges of Severity of Illness (LAPS2) were defined as: Low (0-75), Moderate (75-125), and High (>125), associated with 30-day mortality rates of 2%, 9%, and 30%, respectively.

The rate of transfusion at 24 hours of hospitalization was strongly associated with admission hemoglobin (p < 0.001) (Figure 1A). At 24 hours of hospitalization, 90% of patients with an admission hemoglobin < 7 g/dL received a RBC transfusion, 78% of patients with hemoglobin between 7-7.9 g/dL, 49% of patients with hemoglobin between 8-8.9 g/dL, 19% of those with hemoglobin between 9-9.9 g/dl, and 2% of those with hemoglobin ≥10 g/dl on admission. In general, patients with high severity of illness were not more likely to be transfused than individuals with low and moderate severity of illness. This is evidenced by the clustering of Kaplan-Meier curves stratified by admission hemoglobin (Figure 1B). Small differences in transfusion rates were associated with similar or lower pre-transfusion hemoglobin values in patients with high severity of illness (Additional file 1: Table S1). Similarly, high comorbidity burden was not associated with increased RBC transfusion rates when stratified by admission hemoglobin (Additional file 1: Figure S1). Consistent with this finding, severity of illness (LAPS2) or comorbidity burden (COPS2) scores did not vary by transfusion status, when stratified by admission hemoglobin (Additional file 1: Figure S2).

In general, increased clinical detail yielded better discrimination and explanatory power in modeling RBC transfusion events at 24 hours and through hospitalization (Table 3). For transfusion at 24 hours, the C-statistic for the administrative model was 0.756 and the pseudo-R [2] was 0.196; corresponding values for the comprehensive model were 0.928 and 0.542. For transfusion through the end of hospitalization, the C-statistic for the administrative model was 0.738 and the pseudo-R [2] was 0.176; corresponding values for the comprehensive model were 0.872 and 0.437. A model using admission hemoglobin alone (data not shown) resulted in a C-statistic of 0.842 and 0.736 at 24 hours following admission and through hospitalization, respectively.

**Table 3:** Predictive model performance for red blood cell transfusion

**Model**^**1**^	**Transfusion through 24 hours**		**Ever transfused**	
	**C-statistic**	**Pseudo R**^**2**^	**C-statistic**	**Pseudo R**^**2**^
Administrative data^2^	0.756	0.196	0.738	0.176
(a) + Admission Hemoglobin	0.919	0.522	0.856	0.410
(b) + Severity of Illness	0.922	0.526	0.862	0.418
(c) + Prior RBC Transfusion	0.924	0.530	0.867	0.426
(d) + Initial Hospital Location	0.927	0.537	0.870	0.432
(e) + Hospital	0.928	0.542	0.872	0.437

Footnotes

^1^Model performance in this table is measured using the area under the receiver operator characteristic curve (C-statistic) and Nagelkerke’s Pseudo-R^2^.

^2^Administrative data includes age, sex, comorbid conditions (COPS2), admission type (emergency or elective), and admission diagnosis.

The largest increase in discrimination and explanatory power occurred with the addition of admission hemoglobin (Δ in C-statistic 0.118, IDI 0.344, NRI 1.161) to the model; in contrast comorbidity burden (Δ in C-statistic 0.034, IDI 0.012, NRI 0.299) and severity of illness (Δ in C-statistic 0.006, IDI 0.001, NRI 0.049) had smaller effects. Addition of admission hemoglobin yielded the greatest improvement in statistical performance regardless of the order of introduction into the model relative to other predictors, including severity of illness and comorbidity burden. Complete IDI and NRI results for transfusion through the end of hospitalization in all patients and by clinical diagnoses are shown in Additional file 1: Tables S3-S5.

Table 4 shows model performance throughout hospitalization for the five Primary Conditions. Models including only administrative data, including demographics and comorbidity burden, performed best in patients with malignancy in comparison to those with other diagnoses. Severity of illness (LAPS2) played a relatively small role in improved statistical performance (Δ in C-statistic, IDI, NRI) in all diagnosis-specific conditions. Similar to the cohort as a whole, admission hemoglobin improved statistical performance more than any other factor though less so in patients undergoing orthopedic surgery.

**Table 4:** Predictive model performance for specific medical conditions

**Model**^**1**^	**GI bleed**		**Infection**		**Cardiovascular**		**Malignancy**		**Orthopedic surgery**	
	**N=12,388**		**N=57,473**		**N=47,996**		**N=27,831**		**N=24,264**	
	**C-statistic**	**Pseudo-R**^**2**^	**C-statistic**	**Pseudo-R**^**2**^	**C-statistic**	**Pseudo -R**^**2**^	**C-statistic**	**Pseudo -R**^**2**^	**C-statistic**	**Pseudo-R**^**2**^
Administrative Data^2^	0.587	0.032	0.616	0.038	0.666	0.058	0.828	0.379	0.686	0.121
(a) + Admission Hemoglobin	0.862	0.543	0.839	0.399	0.852	0.419	0.875	0.538	0.696	0.146
(b) + Severity of Illness	0.884	0.566	0.851	0.406	0.866	0.425	0.880	0.547	0.699	0.151
(c) + Prior RBC Transfusion	0.887	0.570	0.862	0.419	0.873	0.432	0.884	0.556	0.709	0.162
(d) + Initial Hospital Location	0.896	0.590	0.871	0.432	0.877	0.440	0.885	0.558	0.710	0.162
(e) + Hospital	0.900	0.599	0.875	0.441	0.884	0.451	0.890	0.563	0.729	0.191

Footnotes

^1^Model performance in this table is measured using the area under the receiver operator characteristic curve (C-statistic) and Nagelkerke’s Pseudo R^2^.

^2^Administrative data includes age, sex, comorbid conditions (COPS2), admission type (emergency or elective), and admission diagnosis.

## Discussion

Using detailed clinical data available prior to hospital admission, we developed a predictive model with very high discrimination for the likelihood of RBC transfusion at 24 hours and through hospitalization. While all predictor variables were statistically significant in all models, the admission hemoglobin was far superior to any other parameter, including comorbidity burden and severity of illness, in predicting the likelihood of RBC transfusion. Administrative data (age, gender, clinical comorbidities, admission type and diagnosis) and the admission hemoglobin alone were sufficient to develop a model with very high calibration, and this finding held true for individual medical conditions such as gastrointestinal bleeding, malignancy, and infection.

It has been hypothesized that the increased severity of illness and complexity of hospitalized patients play a large role in RBC transfusion outside of evidence-based guidelines. Patients with multiple comorbidities or aberrations in vital signs and laboratory values may be seen as having more “physiologic need” for transfusion at lower thresholds of anemia. To date, several studies have examined the role of clinical comorbidities in models of prediction for RBC transfusion. However, these studies have focused exclusively on surgical patients, examining cohorts hospitalized for trauma or preoperatively for cardiac and liver surgery [25-34]. Only studies of trauma examined perioperative changes in severity of illness and found that aberrations in vital signs and laboratory results were predictive of RBC transfusion [28-34]. In this population, even very small changes in heart rate, blood pressure and admission hemoglobin were associated with increased risk of massive transfusion.

The role of severity of illness and clinical comorbidities in predicting RBC transfusion has not been examined in patients hospitalized with common medical conditions. In this study, we utilized advanced measures of comorbid disease burden and severity of illness to risk-adjust patients. We examined the role of these measures, previously validated to assess a patient’s mortality risk, in predicting the likelihood of RBC transfusion events [19]. Given the high acuity of illness in newly admitted patients, we chose to examine RBC transfusion within 24 hours of admission in addition to events through hospitalization. Our results suggest that severity of illness and comorbidity play a relatively minor role in RBC transfusion decisions in the overall hospitalized population.

Our model was less robust in the orthopedic surgery subgroup, compared with other common medical diagnoses that frequently undergo RBC transfusion. This finding is probably due to a larger difference between the admission and pre-transfusion hemoglobin in surgical patients likely due to operative bleeding, which we did not specifically evaluate in this study. Furthermore, perioperative factors such as acute changes in physiology are not reflected in model predictors available at the time of admission. Our reduced ability to discriminate perioperative transfusions is consistent with what has been reported in models of preoperative need for RBC transfusion [25-27]. It is likely that more dynamic scores that factor in trends in vital signs and hemoglobin levels would perform better in predicting the likelihood of RBC transfusion [35].

We were surprised that the predictive ability of severity of illness and clinical comorbidities was so small relative to that of hemoglobin. Overall, patients with high severity of illness or comorbidity burden were not more likely to be transfused than individuals with lower burdens at various admission hemoglobin thresholds. Nor did severity of illness correlate with variation in transfusion practice; pre-transfusion hemoglobin values were similar or lower rather than higher in individuals with high severity of illness. These findings support the notion that clinician’s decision to transfuse RBCs is driven more by a hemoglobin threshold in a particular clinical context (e.g., active bleeding, myocardial ischemia) rather than by a perceived physiologic need of blood based on severity of illness or the degree of clinical comorbidities.

Our data demonstrates a wide range of pre-transfusion hemoglobin levels within and across various admission conditions suggesting that significant variability in transfusion thresholds persists. Several randomized controlled trials in critically ill patients have not supported the role of physiologic need for transfusion above a particular hemoglobin threshold [8,9,36]. In addition to supporting the role for further clinical trials, our observed variability in RBC transfusion for medical and surgical conditions supports an ongoing need for clinician education of evidence-based guidelines. We did observe a higher average hemoglobin trigger for RBC transfusion in cardiovascular and orthopedic conditions compared to that of infection or gastrointestinal bleeding. In some part, these differences may reflect perceived differences in the safety of a restrictive transfusion strategy for particular medical conditions. However, because randomized controlled trial data of transfusion for particular cardiovascular indications such as acute myocardial infarction or septic shock are lacking, equipoise regarding transfusion thresholds for patients with active or underlying cardiovascular disease persists [5,6,8].

Our findings have implications for operational benchmarking and estimating inventory for RBC utilization. For example, one could compare observed rates of transfusion to those expected from the model, by hospital or medical/surgical service, while controlling appropriately for differences in admission hemoglobin and patient characteristics or case mix. Benchmarking could also be used to measure observed versus expected RBC transfusion rates before and after interventions such as implementation of a clinical decision support system. Finally, estimating the likelihood of transfusion based on admission patient characteristics may facilitate blood component management and improve the efficiency of maintaining appropriate blood inventories for hospitalized patients.

A number of limitations of our findings should be stressed. While our patient population is quite relevant in that it reflects the regional community practice of adult inpatients at 21 hospitals in Northern California, it would be desirable to assess this modeling approach in other hospitals, age groups, patient populations and regions of the country. For example, tertiary care referral centers, where trauma and transplant surgery is more common, likely have different patterns of RBC utilization and could require adjustments to the model.

Our model’s ability to explain observed variability in RBC transfusion is high by health services research standards, but opportunities to improve it exist. One current limitation is its lack of data on physician-designated indication for transfusion. Incorporating a clinician’s rationale for transfusion (for example, acute bleeding) or the role of symptoms (chest pain or dyspnea) would add further detail that may further explain practice variation not reflected in the admission or pre-transfusion hemoglobin. With the advent of electronic order entry, clinical decision support systems may allow us to better understand clinician-designated indications for blood transfusion as well as the opportunity to impact current practice.

## Conclusion

Our results help us understand the contribution of various predictors of RBC transfusion at the time of hospitalization in a large cohort of medical and surgical patients. While we identify the importance of the hemoglobin level relative to aberrations in acute physiology and clinical comorbidities, we continue to see significant variability in transfusion practice with a wide range of pre-transfusion hemoglobin for various medical conditions. Future studies examining clinician-designated indications and the role of cardiopulmonary symptoms in the decision to order a transfusion may improve our understanding of current practice and aid in the development of educational interventions to improve compliance with guidelines.

## Competing interest

The authors have no conflicts of interest to disclose relevant to this manuscript.

## Authors’ contributions

NHR had full access to all of the data in the study and takes responsibility for the integrity of the data and the accuracy of the data analysis. Study concept and design: All authors. Acquisition of data: NHR, GJE, BES, MNG. Statistical analysis: NHR, GJE, VL, ELM. Analysis and interpretation of data: All authors. Drafting of the manuscript: NHR, GJE, ELM. Critical revision of the manuscript for important intellectual content: All authors. All authors read and approved the final manuscript.

## Pre-publication history

The pre-publication history for this paper can be accessed here:

http://www.biomedcentral.com/1472-6963/14/213/prepub

## Supplementary Material

Additional file 1 WEB APPENDIX. Click here for file

## **The NHLBI Recipient Epidemiology Donor Evaluation Study - III (REDS-III), Domestic component, is the responsibility of the following persons:**

Hubs:

A.E. Mast and J.L. Gottschall, BloodCenter of Wisconsin (BCW), Milwaukee, WI

D.J. Triulzi and J.E. Kiss, The Institute For Transfusion Medicine (ITXM), Pittsburgh, PA

E.L. Murphy and E.W. Fiebig, University of California, San Francisco (UCSF), San Francisco, CA

E.L. Snyder, Yale University School of Medicine, New Haven, CT and R.G Cable, American Red Cross Blood Services, Farmington CT

Data coordinating center:

D. J. Brambilla and M. T. Sullivan, RTI International, Rockville, MD

Central laboratory:

M.P. Busch and P.J. Norris, Blood Systems Research Institute, San Francisco, CA

Publication Committee Chairman:

R. Y. Dodd, American Red Cross, Holland Laboratory, Rockville, MD

Steering Committee Chairman:

S. H. Kleinman, University of British Columbia, Victoria, BC, Canada

National Heart, Lung, and Blood Institute, National Institutes of Health:

S. A. Glynn and A.M. Cristman
