# Supplementary material for: Predicting red blood cell transfusion in hospitalized patients: role of hemoglobin level, comorbidities, and illness severity
Source: BMC Health Serv Res. 2014 May 10;14:213. doi: 10.1186/1472-6963-14-213 (PMC4101854; doi:10.1186/1472-6963-14-213)
Supplement: Additional file 1 — WEB APPENDIX. [file 1472-6963-14-213-S1.docx]

**Predicting Red Blood Cell Transfusion in Hospitalized Patients: Role of Hemoglobin Level, Comorbidities, and Illness Severity**

Nareg H. Roubinian, Edward L. Murphy, Bix E. Swain, Marla N. Gardner, Vincent Liu, Gabriel J. Escobar

**WEB APPENDIX**

**Number Description Pages**

1 Methods 1 - Audit of blood product transfusion record 2

2 Methods 2 - Grouping of HCUP Codes into Primary Conditions 3

3 Appendix Table 1 4

4 Appendix Table 2 5

5 Appendix Table 3 6

6 Appendix Table 4 7

7 Appendix Table 5 8

8 Appendix Figure 1 9

9 Appendix Figure 2 10

10 References 11

**Appendix Methods 1: Audit of Kaiser Permanente Northern California Blood Product Record**

Our team performed multiple initial audits to develop the data processing strategy used in the development of the merged inpatient transfusion dataset. To validate the accuracy of the Kaiser Permanente Northern California (KPNC) blood bank database, a trained research nurse audited a random sample of 600 charts of adult patients using electronic medical records. The research nurse reviewed data from progress notes, operative notes, nursing flowsheets, and discharge summaries where these were available. The blood bank database included records of allogeneic as well as pre-operative autologous blood products but did not include intra or post-operative cell salvage transfusions.

This audit included chart reviews divided between verifying administration of blood products when noted in the blood bank database as well as to verify that non-transfused patients indeed did not receive a transfusion during their hospital stay. For each audit, 300 patient hospitalizations were reviewed and divided into groups of 100 for examination of red blood cell, platelet, and fresh frozen plasma transfusion.

No record of blood product transfusion (0/300 hospitalizations) was identified that was not noted in the blood bank database, however, four instances of post-operative cell salvage transfusion, which are not recorded by the blood bank, were identified. A transfusion event could not be verified in 10 hospitalizations (290/300 transfusion events identified). In 9 of these hospitalizations documentation was limited due to timing of implementation of electronic medical records, and transfusion was not able to be verified. In the remaining hospitalization with full electronic documentation, no record of blood product transfusion was identified.

**Appendix Methods 2: Grouping of HCUP Codes into Primary Conditions**

Primary Conditions are groupings of related International Classification of Disease codes assigned at the time of admission to the hospital. The codes are grouped based on the schema used by the Agency for Healthcare Research and Quality’s Healthcare Cost & Utilization Project (HCUP). The HCUP single-level diagnosis categories were grouped based on biologic plausibility (i.e., relative similarity from a disease standpoint) and by common indications for admission, association with bleeding, or an increase utilization of blood products. These groupings included: Gastrointestinal Bleeding, Malignancy, Cardiovascular, Infection, and Orthopedic Surgery. The grouping for gastrointestinal bleeding is based upon HCUP single-level diagnosis code 153 which includes admission ICD-9 codes for upper and lower gastrointestinal bleeding (e.g., peptic ulcer, cirrhosis, diverticulosis, etc). The grouping for Cardiovascular conditions included non-surgical admission diagnoses for acute myocardial infarction, coronary atherosclerosis, valvular disorders, congestive heart failure, dysrhythmias, peripheral vascular disease, and pulmonary heart disease (HCUP Codes 96, 100, 101, 102, 103, 106, 108, and 114). The grouping for Malignancy included admission diagnoses for conditions related to cancer including elective chemotherapy (HCUP Codes 11 through 47 and 63). The grouping for Infection included admission diagnoses for sepsis, pneumonia, urinary tract infections, lower tract respiratory disease, and skin tissue infection (HCUP Codes 20, 122, 159, 133, and 197 respectively). The grouping for Orthopedic Surgery included surgical admissions for orthopedic fracture or osteoarthritis (HCUP Code 203, 207, and 226).

**Appendix Table 1 - Pre-Transfusion Hemoglobin Stratified by Severity of Illness & Admission Hemoglobin**

| **Severity of Illness^1^** | **Admission Hemoglobin** | | | | |
| --- | --- | --- | --- | --- | --- |
|  | **< 7.0 g/dL** | **7-7.9 g/dL** | **8.0-8.9 g/dL** | **9.0-9.9 g/dL** | **> 10 g/dL** |
|  | Pretransfusion Hemoglobin^2^ | Pre-transfusion Hemoglobin^2^ | Pre-transfusion Hemoglobin^2^ | Pre-transfusion Hemoglobin^2^ | Pre-transfusion Hemoglobin^2^ |
| Low | 5.9 ± 1.0 | 7.4 ± .63 | 8.1 ± .46 | 8.4 ± .91 | 8.9 ± 1.58 |
| Moderate | 5.9 ± 1.1 | 7.5 ± .57 | 8.0 ± .44 | 8.3 ± .84 | 8.7 ± 1.28 |
| High | 6.0 ± 1.2 | 7.5 ± .66 | 8.0 ± .81 | 8.2 ± .95 | - 1. ± 1.28 |
| Combined | 5.9 ± 1.1 | 7.4 ± .61 | 8.0 ± .72 | 8.3 ± .87 | 8.7 ± 1.46 |

Footnotes

1. Severity of Illness refers to ranges of Laboratory Acute Physiology Score, version 2 (LAPS2) a physiology-based score which includes vital signs, neurological status, and laboratory results^1^. Increasing degrees of physiologic derangement and are reflected in a higher LAPS2.

Ranges of Severity of Illness (LAPS2) were defined as: Low (0-75), Moderate (75-125), and High (>125). Low, Moderate, and High Severity of Illness were associated with 30-day mortality rates of 2%, 9%, and 30%, respectively.

1. First Pre-transfusion hemoglobin (mean in g/dL ± SD)

**Appendix Table 2: Model Performance for RBC Transfusion through 24 Hours of Hospitalization**

|  | **GI Bleed N=12,388** | | | **Infection N=57,473** | | | **Cardiovascular N= 47,996** | | **Malignancy N= 27,831** | | **Orthopedic Surgery**  **N= 24,264** | |
| --- | --- | --- | --- | --- | --- | --- | --- | --- | --- | --- | --- | --- |
| **Model^1^** | | C-Statistic | Pseudo-R^2^ | | C-Statistic | Pseudo-R^2^ | C-Statistic | Pseudo -R^2^ | C-Statistic | Pseudo -R^2^ | C-Statistic | Pseudo-R^2^ |
| **Administrative Data^2^** | | 0.575 | 0.024 | | 0.617 | 0.027 | 0.667 | 0.048 | 0.857 | 0.423 | 0.709 | 0.085 |
| **(b) + Admission Hemoglobin** | | 0.879 | 0.591 | | 0.929 | 0.539 | 0.939 | 0.581 | 0.901 | 0.609 | 0.736 | 0.155 |
| **(c) + Severity of Illness** | | 0.899 | 0.610 | | 0.932 | 0.540 | 0.940 | 0.581 | 0.906 | 0.615 | 0.736 | 0.155 |
| **(c) + Prior RBC Transfusion** | | 0.901 | 0.612 | | 0.936 | 0.545 | 0.943 | 0.585 | 0.909 | 0.622 | 0.741 | 0.158 |
| **(d) + Initial Hospital Location** | | 0.911 | 0.636 | | 0.941 | 0.554 | 0.945 | 0.590 | 0.910 | 0.623 | 0.747 | 0.165 |
| **(e) + Hospital** | | 0.916 | 0.647 | | 0.943 | 0.563 | 0.949 | 0.600 | 0.913 | 0.627 | 0.812 | 0.228 |

Footnotes:

1. Model performance in this table is measured using the area under the receiver operator characteristic curve (C-statistic) and Nagelkerke’s Pseudo-R2.
2. Administrative data includes age, sex, comorbid conditions (COPS2), admission type (emergency or elective), and admission diagnosis

**Appendix Table 3: Δ in C-Statistic, IDI & NRI for All Patients through Hospitalization**

| **Model^1^** | **Δ in C-Statistic** | **Integrated Discrimination Improvement** | **Net Reclassification Improvement** |
| --- | --- | --- | --- |
| **(a) Administrative Data excluding Comorbidities^2^** |  |  |  |
| **(b) Administrative Data including Comorbidities^2^** | 0.034 | 0.0125 | 0.2986 |
| **(c) (b) + Admission Hemoglobin** | 0.118 | 0.3444 | 1.1609 |
| **(d) (c) + Severity of Illness** | 0.006 | 0.0003 | 0.0488 |
| **(e) (d) + Prior RBC Transfusion** | 0.005 | 0.0039 | 0.0184 |
| **(f) (e) + Initial Hospital Location** | 0.003 | 0.0005 | 0.1008 |

Footnotes:

1. Model performance in this table is measured using the area under the receiver operator characteristic curve (C-statistic), Integrated Discrimination Improvement, and Net Reclassification Improvement.^23^
2. Administrative data includes age, sex, comorbid conditions (COPS2), admission type (emergency or elective), and admission diagnosis

|  | **Appendix Table 4: Integrated Discrimination Index for Specific Medical Conditions through Hospitalization** | | | | | | |
| --- | --- | --- | --- | --- | --- | --- | --- |
| **Model** | | **GI Bleed** | **Infection** | **Cancer** | **Cardiovascular** | **Orthopedic Surgery** |  |
| **(a) Administrative Data excluding Comorbidities^1^** | |  |  |  |  |  |  |
| **(b) Administrative Data including Comorbidities^1^** | | 0.0150 | 0.0125 | 0.0138 | 0.0145 | 0.0076 |  |
| **(c) (b) + Admission Hemoglobin** | | 0.4826 | 0.3302 | 0.2983 | 0.3288 | 0.1733 |  |
| **(d) (c) + Severity of Illness** | | 0.0119 | 0.0076 | 0.0021 | 0.0000 | 0.0001 |  |
| **(e) (d) + Prior RBC Transfusion** | | 0.0563 | 0.0061 | 0.0031 | 0.0028 | 0.0011 |  |
| **(f) (e) + Initial Hospital Location** | | 0.3686 | 0.0022 | 0.0001 | 0.0018 | 0.0002 |  |

Footnotes:

1. Administrative data includes age, sex, comorbid conditions (COPS2), admission type (emergency or elective), and admission diagnosis

| **Appendix Table 5: Net Reclassification Improvement for Specific Medical Conditions through Hospitalization** | | | | | |  |
| --- | --- | --- | --- | --- | --- | --- |
| **Model** | **GI Bleed** | **Infection** | **Cancer** | **Cardiovascular** | **Orthopedic Surgery** | |
| **(a) Administrative Data excluding Comorbidities^1^** |  |  |  |  |  | |
| **(b) Administrative Data including Comorbidities^1^** | 0.2331 | 0.2937 | 0.2049 | 0.4101 | 0.2578 | |
| **(c) (b) + Admission Hemoglobin** | 1.3222 | 1.1877 | 1.2452 | 1.1939 | 0.7629 | |
| **(d) (c) + Severity of Illness** | 0.2958 | 0.2853 | 0.2309 | 0.1168 | 0.0468 | |
| **(e) (d) + Prior RBC transfusion** | 0.0563 | 0.0918 | 0.1965 | 0.1327 | 0.0420 | |
| **(f) (e) + Initial Hospital Location** | 0.3686 | 0.2778 | 0.7728 | 0.1475 | 0.1121 | |

Footnotes:

1. Administrative data includes age, sex, comorbid conditions (COPS2), admission type (emergency or elective), and admission diagnosis


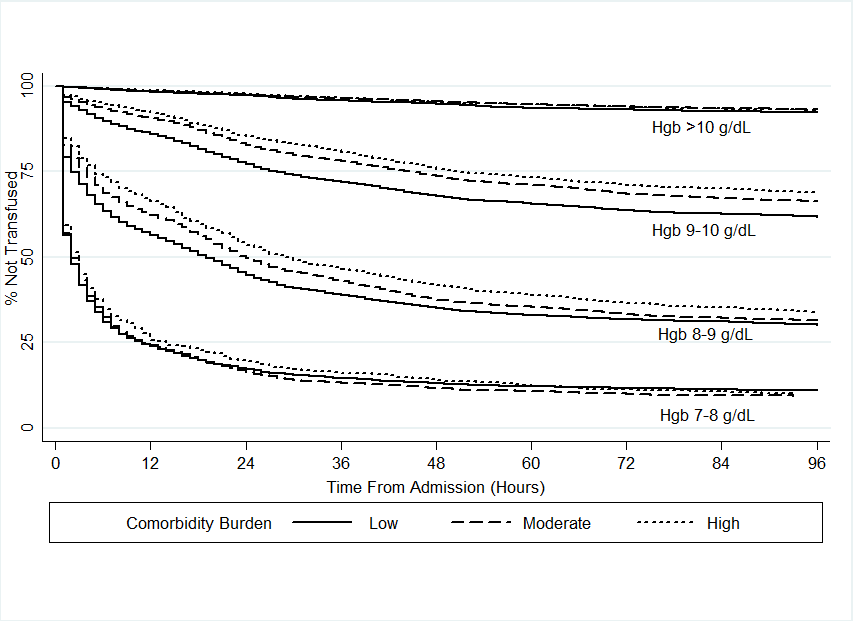


**Appendix Figure 1 - Probability of Red Blood Cell Transfusion as a Function of Comorbidity Burden**

The right panel shows that increasing comorbidity burden, within varying strata of admission hemoglobin, does not explain differences in overall rates of transfusion.

Comorbidity burden was classified by a previously described continuous score, COPS2, which is based upon patients’ medical diagnoses for the 12 months preceding hospitalization.^1^

Ranges of Comorbidity Burden (COPS2) were defined as: Low (0-75), Moderate (75-125), and High (>125). Low, Moderate, and High Comorbidity Burden were associated with 30-day mortality rates of 4%, 11%, and 14%, respectively.


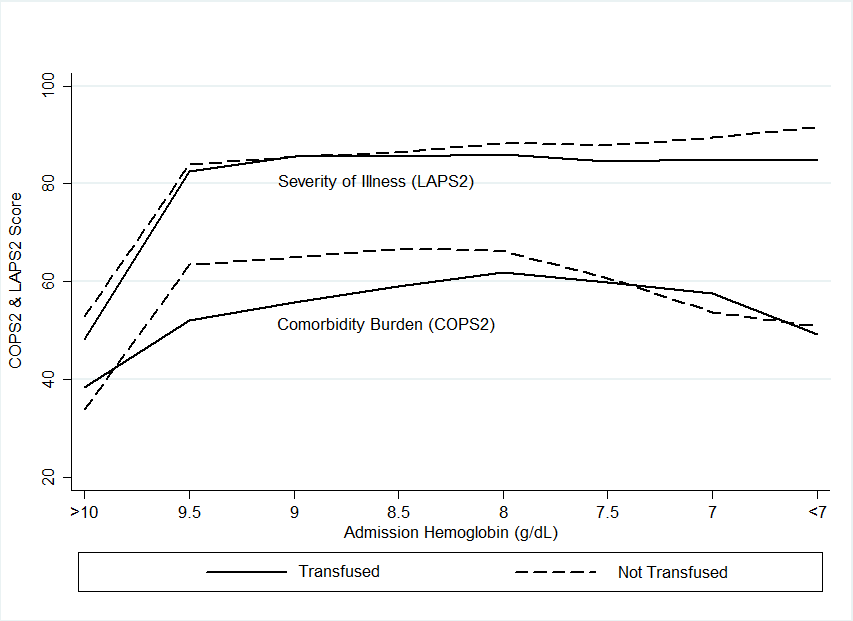


**Appendix Figure 2 – Comorbidity Burden & Severity of Illness by Transfusion Status**

The figure shows that for progressively increasing degrees of anemia on admission, comorbidity burden (COPS2) and severity of illness (LAPS2) were similar between transfused and non-transfused patients in the first 24 hours of admission.

References

1. Escobar GJ, Gardner MN, Greene JD, Draper D, Kipnis P. Risk-adjusting hospital mortality using a comprehensive electronic record in an integrated healthcare delivery system. Medical Care, May 2013;51(5):446-53.
